# Plasma acetylcholine and nicotinic acid are correlated with focused preference for photographed females in depressed males: an economic game study

**DOI:** 10.1038/s41598-020-75115-4

**Published:** 2021-01-26

**Authors:** Hiroaki Kubo, Daiki Setoyama, Motoki Watabe, Masahiro Ohgidani, Kohei Hayakawa, Nobuki Kuwano, Mina Sato-Kasai, Ryoko Katsuki, Shigenobu Kanba, Dongchon Kang, Takahiro A. Kato

**Affiliations:** 1 Department of Neuropsychiatry, Graduate School of Medical Sciences, Kyushu University, 3-1-1 Maidashi Higashi-Ku, Fukuoka, 812-8582 Japan; 2 Department of Clinical Chemistry and Laboratory Medicine, Graduate School of Medical Sciences, Kyushu University, 3-1-1 Maidashi Higashi-Ku, Fukuoka, 812-8582 Japan; 3 School of Business, Monash University Malaysia, Jalan Lagoon Selatan, 46150 Bandar Sunway, Selangor Darul Ehsan Malaysia

**Keywords:** Psychology, Biomarkers, Medical research

## Abstract

Interpersonal difficulties are often observed in major depressive disorder (MDD), while the underlying psychological and biological mechanisms have not yet been elucidated. In the present case–control study, a PC-based trust game was conducted for 38 drug-free MDD patients and 38 healthy controls (HC). In the trust game, participants invested money in a partner (trusting behaviors), and also rated each partner’s attractiveness (preference for others). In addition, blood biomarkers including metabolites were measured. Both MDD and HC males exhibited more trusting behaviors compared to females. MDD males’ preference for ordinary-attractive partners (lay-person photographs) was lower than HC males, whereas their preference for high-attractive females (fashion-model photographs) was similar levels to HC males. This tendency in MDD males could reflect a “focused (narrowed) preference for females”. As for blood biomarker analysis, the levels of 37 metabolites including acetylcholine, AMP, GMP, nicotinic acid and tryptophan were significantly different between two groups. Interestingly, among male participants, acetylcholine and nicotinic acid were negatively correlated with the level of focused preference for photographed females. In sum, we have revealed some behavioral, psychological and biological traits of trusting behaviors and preference for others especially in MDD males. Larger studies should be conducted to validate our preliminary findings.

## Introduction

Major depressive disorder (MDD) is one of the most common psychiatric conditions observed worldwide^1–3^. MDD patients show not only depressive feelings but also loss of interests, difficulties in social interaction and cooperative behavior^4^. A previous study showed that low interpersonal trust was associated with both new-onset and long-term depression^5^. Therefore, exploring interpersonal aspects including trust is warranted to elucidate the pathology of MDD. Decision-making tasks have been highlighted in psychiatry as a method to understand the deeper behavioral traits beyond evaluating patient self-reported symptoms^6–10^. Trust game is a novel approach to investigate trusting behaviors, and is a kind of economic game developed in the field of social psychology and economics^11^. Trust game has been widely used to examine psychiatric disorders such as schizophrenia, borderline personality disorder, and depression^12–16^. Previous reports have shown some correlations between depressive symptoms and trusting behaviors using trust game^17–20^. Furthermore, recent studies, using trust game, have paid much attention to the biological basis of trust, indicating that oxytocin and testosterone influence trusting behaviors^21–25^.

On the other hand, blood metabolites have been suggested as a candidate objective biomarker of depression^26–32^. We have developed an original PC-based trust game, which evaluates not only trustworthiness to others but also preference for others using photographed partners’ faces with different levels of attractiveness^19,33–36^. Using the PC-based trust game, our previous research targeting university students has revealed that not only trusting behaviors (monetary scores invested to the partners) but also preference for partners (rating scores of the partners’ attractiveness) were associated with blood biomarkers such as serum high density lipoprotein cholesterol (HDL-C), uric acid (UA), high sensitivity C-reactive protein (hsCRP) and plasma fibrin degeneration products (FDP)^36^.

Loss of interest/pleasure is a crucial symptom of depression^4,37,38^. However, this symptom is usually assessed in accordance with subjective information provided by patients and objective evaluation tools are not yet established. Our previous study using the PC-based trust game with photographed partners’ faces has shown that trusting behaviors are influenced by attractiveness of the partners’ faces (Watabe et al. PLOS ONE 2015). However, it remains unclear how the attractiveness of the partners influences trusting behavior in patients with depressed individuals. We hypothesize that loss of interest/pleasure may alter attractiveness-driven behaviors in MDD patients. We expect that our PC-based trust game would be an appropriate tool to grasp such aspects by evaluating trusting behaviors and preference for others, both of which are relevant to pro-social and motivational aspects. Therefore, the aim of the present case–control study is to investigate MDD patients’ trusting behaviors and preference for others using our PC-based trust game, and is to explore the biological basis by evaluating blood biomarkers.

## Methods

This case–control study was approved by the ethics committee of Kyushu University (30-452) and was carried out in accordance with the Declaration of Helsinki.

### Participants

Drug-free MDD patients and healthy subjects (age and gender matched controls) were enrolled in the present study as participants. All participants were Japanese (Asian) and provided their written informed consent prior to the study. Patients were recruited at Kyushu University Hospital and its related affiliations (mainly outpatient clinics). Healthy controls were recruited via flyers on Kyushu University Hospital and university campus. Structured Clinical Interview for DSM-IV-TR (SCID)^39^ was conducted by trained psychiatrists for the diagnosis of MDD. 38 drug-free MDD patients (22 males and 16 females) were selected for the present study. As exclusion criteria for MDD patients, we confirmed that all the patients had no history of the following diseases; neurodegenerative diseases, psychotic disorders, mental retardation, substance abuse, and physical diseases such as cardiovascular diseases, liver and kidney diseases, infectious diseases, malignant tumors, and head trauma. 38 healthy subjects were selected as healthy controls (HC) by interviews based on the SCID regarding any previous or ongoing psychiatric disorders, physical diseases, and medications, which were set as exclusion criteria.

### Procedure

All participants completed a series of interviews and self-rated questionnaires, played a PC-based trust game, and provided a non-fasting venous blood sample.

### Interview

SCID was conducted for all the participants. Severity of depression was assessed with the 17-item Hamilton Rating Scale for Depression (HAMD-17) by trained psychiatrists or clinical psychologists^38,40^.

### Self-rated questionnaires

A Self‐report questionnaire evaluating the severity of depression was implemented using the Japanese version of Beck Depression Inventory-Second Edition (BDI-II)^41^. In addition, Yamagishi and Yamagishi’s trust scale (YYS) was conducted to measure respondents’ estimation of others’ trustworthiness^42,43^. Furthermore, 140-item Temperament and Character Inventory (TCI-140) was conducted to measure seven basic personality dimensions (four temperaments; Novelty Seeking, Harm Avoidance, Reward Dependence, Persistence, and three characters; Self-Directedness, Cooperativeness, Self-Transcendence)^44,45^.

### PC-based trust game

As introduced in our previous reports^19,36^, we have developed an original PC-based trust game, which was conducted using a laptop computer to evaluate both participants’ trusting behaviors and preference for others. Participants (trustors) were instructed on the game rules. Then, the participants were required to make decisions regarding how much of 1300 JPY (about 12 USD) to give to each of 40 partners (trustees) and also to rate the partners’ attractiveness based on facial photographs presented on a computer screen (the score ranges: 0–9), which is thought to reflect raters’ subjective preference for others (Preference Score). The amount of money given to the partner by the participant (Monetary Score) is tripled, and the partner then decided whether to split the money equally with the participant or take the entire amount of the money. The participant’s decision as to how much money to give to the partner is thought to reflect their level of trust in the partner.

In this experiment, partners were virtual players on a computer screen. The participants had no information about their partners except for the facial photographs including the head and shoulders with a neutral facial expression. Photos of the partners were selected from professional fashion models (i.e., “high-attractive partners”) or lay individuals (i.e., “ordinary-attractive partners”). Regarding the photographs of lay individuals, photographs were originally taken for our game development with permission. We randomly selected 20 pictures (10 males and 10 females) to use in the present experiment. We also added 20 pictures of fashion models (10 males and 10 females) taken from a DVD with the same composition under permission by the publisher. This diversity of physical attractiveness was expected to have an effect on decisions in the trust game. All photographs are of Japanese. The participant was not aware of the partner’s decision. After the experiments, each participant was actually paid the amount of money corresponding to the result of a randomly selected game from all games as a reward.

### Blood biomarkers

All peripheral venous blood samples were collected between 10:00 and 15:00. The plasma was immediately extracted and then frozen and stored at − 80 °C until required for analysis. Blood biomarkers measured included routine blood biochemical markers and blood metabolites (Metabolomics).

Routine blood biochemical markers including serum total-cholesterol (Total-C), high density lipoprotein-cholesterol (HDL-C), low density lipoprotein-cholesterol (LDL-C), fibrinogen (Fib), fibrin/fibrinogen degradation products (FDP), total-bilirubin (T-bil), direct-bilirubin (D-bil), indirect-bilirubin (I-bil), uric acid (UA), and high-sensitivity C-reactive protein (hsCRP) were measured by automatic biochemical analyzer (SRL, Inc., Tokyo, Japan). Plasma metabolites were measured by liquid chromatography–mass spectrometry (LC–MS) using LCMS-8060 (Shimadzu Corp., Kyoto, Japan), as described previously^26^.

### Statistics

We first performed correlation analysis to assess monotonous relationships between monetary score, preference score, and self-rated questionnaires (YYS and each sub-scale of TCI-140). Pearson's product-moment correlation coefficient was calculated for normal distribution data; Spearman's rank correlation coefficient was calculated for non-normal distribution data, respectively.

Next, we performed ANOVA. Independent variables were participants’ gender (male, female) and psychiatric condition (MDD, HC). Dependent variables were monetary score and preference score for partners presented in the PC-based trust game. ANOVA was performed at four types of photographed partners (ordinary-attractive males, ordinary-attractive females, high-attractive males, and high-attractive females), respectively.

Then, we measured routine blood biochemical markers and plasma metabolites, and subsequent group comparison (MDD vs HC) was performed using Mann–Whitney U test.

## Results

Demographics and clinical variables are summarized in Table 1. There was no significant difference in age between MDD patients and HC participants. MDD patients’ trust toward others assessed with a self-rated questionnaire (YYS) was significantly lower than that of HC participants. As for TCI-140 sub-scales, Harm Avoidance was significantly higher and Self-Directedness was lower in MDD than HC, which was consistent with previous reports^46–48^.

**Table 1:** Demographics and clinical variables of drug-free MDD patients and Healthy Controls (HC).

	Male		Female		p value (2-way ANOVA) Main effect of condition Main effect of gender Interaction
	MDD	HC	MDD	HC	
N	22	22	16	16	–
Age	30.41	29.36	30.25	31.63	0.99 0.55 0.49
SD	6.59	6.09	10.32	5.89	
HAM-D	18.82	1.32	16.88	1.56	0.00*** 0.37 0.25
SD	6.20	1.52	3.62	2.03	
BDI-II	32.27	6.27	34.27	4.56	0.00*** 0.96 0.36
SD	11.56	7.36	5.48	5.87	
YYS	22.33	27.91	22.47	26.75	0.00*** 0.70 0.64
SD	6.62	4.25	4.35	6.73	
**TCI-140**					
Novelty seeking	56.90	60.50	54.07	63.13	0.00** 0.99 0.15
SD	6.48	9.91	8.73	6.11	
Harm avoidance	74.62	59.59	73.53	63.88	0.00*** 0.56 0.35
SD	14.38	9.34	14.67	9.75	
Reward dependence	59.76	65.95	63.53	72.19	0.00** 0.02* 0.57
SD	10.46	9.22	9.99	6.20	
Persistence	48.10	61.59	58.87	60.81	0.00*** 0.06† 0.02*
SD	10.09	11.25	12.29	8.50	
Simple main effect (Persistence)		MDD male < HC male (*p* < 0.01) MDD male < MDD female (*p* < 0.01)			
Self-Directedness	50.29	67.68	54.14	67.69	0.00*** 0.48 0.46
SD	11.82	12.52	8.04	8.58	
Cooperativeness	66.10	70.95	68.79	71.81	0.07† 0.44 0.68
SD	9.67	8.99	10.16	8.77	
Self-Transcendence	32.14	31.14	35.87	34.38	0.57 0.11 0.91
SD	10.72	5.76	8.23	11.31	

^†^*p* < 0.10, *p < 0.05, **p < 0.01, ***p < 0.001.

### Monetary score (trusting behaviors)

To evaluate trusting behaviors, a PC-based trust game including four types of photographed partners was conducted for MDD patients and HC participants. Correlation of monetary score with YYS and TCI-140 sub-scales was shown in Supplementary Table S1. ANOVA of monetary score for four types of photographed partners was shown in Table 2.

**Table 2:** Monetary score (trusting behaviors) for each type of photographed partner.

	Male		Female		p value (2-way ANOVA) Main effect of condition Main effect of gender Interaction
	MDD	HC	MDD	HC	
Ordinary-attractive males	503.79	622.35	339.05	444.27	0.16 0.03* 0.93
SD	411.58	320.37	311.28	276.35	
Ordinary-attractive females	445.00	605.00	334.38	447.50	0.08† 0.09† 0.76
SD	392.28	309.67	320.06	277.74	
High-attractive males	397.73	572.73	273.44	350.78	0.09† 0.02* 0.51
SD	388.84	286.44	299.09	240.46	
High-attractive females	462.27	604.54	338.74	415.63	0.15 0.04* 0.66
SD	358.14	300.21	307.55	303.91	

^†^*p* < 0.10, *p < 0.05.

### Correlation of monetary score (Supplementary Table S1)

YYS score was positively correlated with monetary score in HC, whereas the correlation was not significant in MDD patients. As for TCI-140 sub-scales, Reward Dependence has shown significantly positive correlation with monetary score in HC males.

### ANOVA of monetary score for four types of photographed partners (Table 2)

As a whole, both MDD and HC males showed greater trusting behaviors toward partners compared to females. Moreover, MDD patients tended to invest less money in ordinary-attractive photographed female partners and high-attractive male partners.

### Preference score

In the trust game, preference score was measured by requiring participants to rate the partners’ attractiveness within a 10-point rating (0–9). Correlation of preference score with YYS and TCI-140 sub-scales was shown in Supplementary Table S1. ANOVA of preference score for four types of photographed partners was shown in Table 3.

**Table 3:** Preference score for each type of photographed partner.

	Male		Female		p value (2-way ANOVA) Main effect of condition Main effect of gender Interaction
	MDD	HC	MDD	HC	
Ordinary-attractive males	2.31	3.72	2.52	2.64	0.04* 0.23 0.08^†^
SD	1.79	1.58	1.14	1.56	
Simple main effect		MDD male < HC male (*p* < 0.01)			
Ordinary-attractive females	1.97	3.38	2.68	2.82	0.03* 0.84 0.08^†^
SD	1.83	1.41	1.29	1.33	
Simple main effect		MDD male < HC male (*p* < 0.01)			
High-attractive males	3.09	4.48	2.71	3.29	0.01** 0.04* 0.28
SD	1.92	1.37	1.30	1.65	
High-attractive females	4.05	4.85	3.94	4.31	0.14 0.40 0.58
SD	1.88	1.23	1.64	1.83	

^†^*p* < 0.10, *p < 0.05, **p < 0.01.

### Correlation of preference score (Supplementary Table S2)

YYS score was positively correlated with preference score in HC males, HC females and MDD females; whereas the correlation was weaker in MDD males. As for TCI-140 sub-scales, Reward Dependence and Cooperativeness were positively correlated with preference score in HC males.

### ANOVA of preference score for four types of photographed partners (Table 3)

For ordinary-attractive partners, MDD males’ preference score was lower than that of HC males. On the other hand, surprisingly, MDD males’ preference score for high-attractive female partners was similar levels to HC males.

### Focused preference index

**Figure 1:**
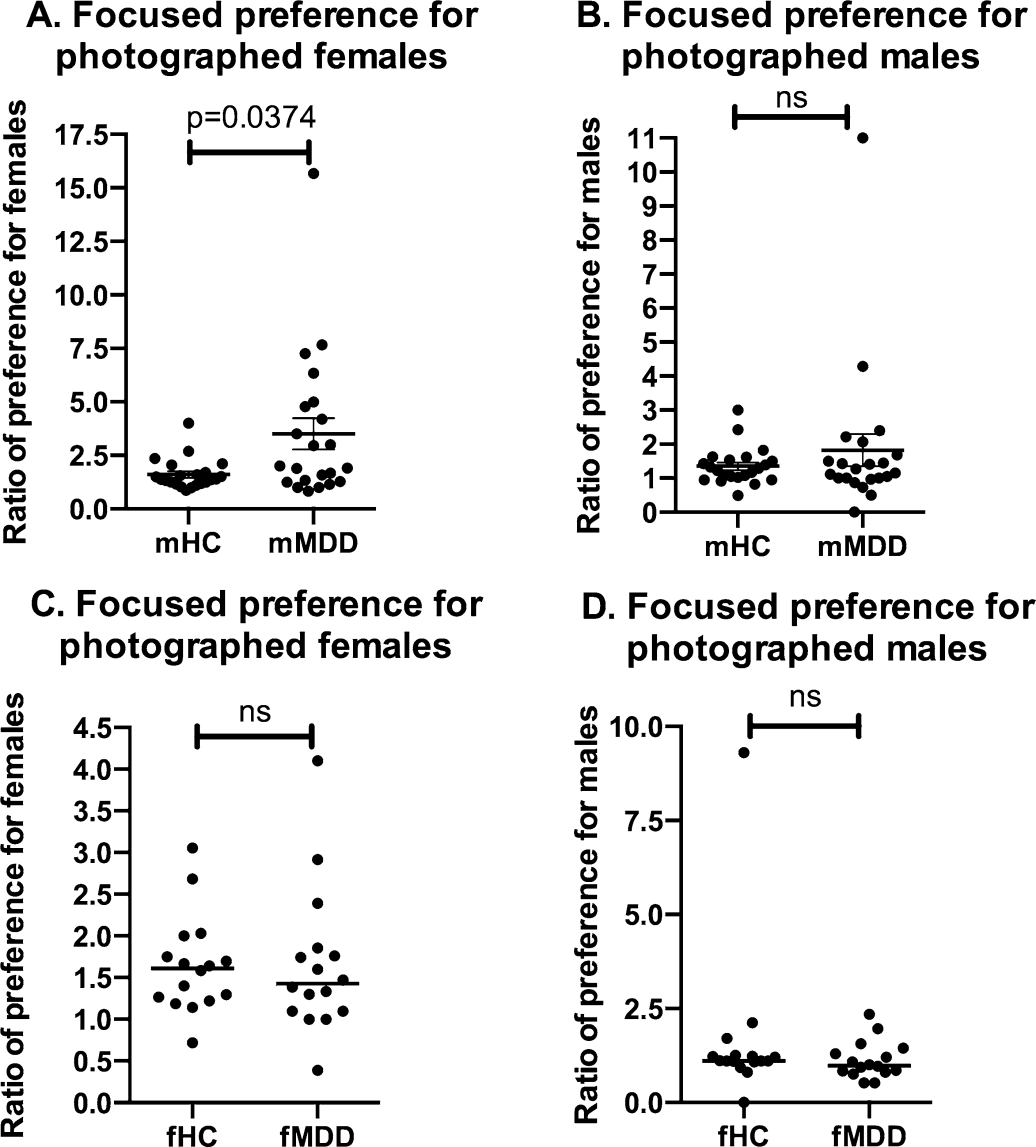
Group comparison of focused preference index for photographed partners. Dot plots show differences in ratio of preference for male and female partners (i.e. ration of focused preference index) between HC and MDD males (**A**, **B**), and HC and MDD females (**C**, **D**). Mann–Whitney's U test was performed for group comparison, and MDD males’ focused preference was higher than HC males (**A**). Horizontal bars represent the median values of each group. Male HC (mHC): N = 22; male MDD (mMDD): N = 22; female HC (fHC): N = 16; female MDD (fMDD): N = 16. *HC* healthy controls, *MDD* major depressive disorder.

This trait of MDD males might be caused by focused (or narrowed) preference. Thus, we defined the ratio of the mean preference score for ordinary-attractive partners to that of high-attractive partners as “focused preference index.” This index is higher when the mean preference score of ordinary-attractive partner is low and that of high-attractive partner is high. Scores of focused preference index were shown in Supplementary Table S3. Mann–Whitney U test was performed to investigate the focused preference for photographed partners (shown in Fig. 1 and Supplementary Table S3). Interestingly, MDD males’ focused preference for photographed females was higher than that of HC males (Fig. 1A). On the other hand, focused preference for photographed males was not significantly different between MDD and HC males (Fig. 1B). These results indicate that MDD males’ preference is focused (narrowed), especially for photographed females.

### Group comparison of biomarkers between MDD and HC

Plasma metabolome analysis was performed for all subjects, and 71 metabolites were successfully identified (shown in Supplementary Table S4). We also measured 11 routine blood biochemical markers related to lipids, blood coagulation, and inflammation (Total-C, HDL-C, LDL-C, Fib, FDP, T-Bil, D-Bil, I-Bil, UA, and hsCRP) based on our previous studies^26,36^ (shown in Supplementary Table S5). A comparison of the above blood biomarkers between MDD patients and HC participants showed significant differences among 33 metabolites including acetylcholine, adenosine monophosphate (AMP) and guanosine monophosphate (GMP), nicotinic acid and tryptophan (Table 4 and Supplementary Table S4), and 4 routine blood biochemical markers especially bilirubin (Supplementary Table S5).

**Table 4:** Significantly different blood biomarkers in MDD and HC.

Characteristic^a^	MDD, N = 38	HC, N = 38	p-value
Acetylcholine	525 (226) ↓	709 (236)	< 0.001***
Aconitic acid	4483 (1308) ↓	5056 (1359)	0.032*
Adenosine	6457 (1704) ↓	8595 (3352)	< 0.001***
Adenosine monophosphate	10,650 (7802) ↓	24,593 (14,547)	< 0.001***
Alanine	99,181 (38,100) ↓	117,996 (42,002)	0.022*
Arginine	54,014 (29,684) ↓	129,668 (81,167)	< 0.001***
Asymmetric dimethylarginine	2813 (766) ↓	3191 (701)	0.014*
Carnitine	61,205 (23,678) ↓	72,086 (24,752)	0.027*
Creatinine	97,260 (31,705) ↓	117,191 (43,397)	0.013*
Cystine	5208 (3600) ↓	15,821 (11,160)	< 0.001***
Cytidine	496 (201) ↓	586 (182)	0.022*
Dimethylglycine	10,777 (3951) ↓	13,518 (5389)	0.007**
Fumaric acid	813 (251) ↑	684 (233)	0.011*
Glutamine	518,638 (168,131) ↓	599,275 (191,132)	0.027*
Glycine	6231 (2915)	8376 (3145)	0.001**
Guanosine	3081 (851) ↓	3683 (817)	0.001**
Guanosine monophosphate	1223 (910) ↓	2162 (1328)	< 0.001***
Histidine	130,431 (49,268) ↓	167,986 (56,561)	0.001**
Inosine	628 (386) ↓	1091 (1646)	0.050*
2-Ketoglutaric acid	21,940 (2955) ↓	25,145 (3029)	< 0.001***
Lactic acid	126,455 (44,543) ↑	68,039 (57,343)	< 0.001***
Lysine	561,157 (185,821) ↓	639,687 (201,374)	0.041*
Methionine	7639 (3303) ↓	12,849 (4486)	< 0.001***
Nicotinic acid	1986 (489) ↓	2325 (434)	0.001**
Norepinephrine	8848 (3006) ↓	10,884 (3327)	0.003**
Ornitine	118,005 (40,153) ↑	89,556 (53,588)	0.005**
Orotic acid	11,336 (6503) ↑	4986 (1514)	< 0.001***
Pyruvic acid	1089 (404) ↑	754 (511)	0.001**
Serine	44,256 (20,273) ↓	53,241 (20,214)	0.028*
Succinic acid	710 (389) ↑	454 (231)	< 0.001***
Symmetric dimethylarginine	3494 (1137) ↓	3966 (802)	0.025*
Tryptophan	65,924 (26,140) ↓	78,356 (25,559)	0.020*
Tyrosine	28,388 (11,153) ↓	33,175 (12,407)	0.041*

^a^Statistics presented: mean of signal intensity (SD). ^†^*p* < 0.10, *p < 0.05, **p < 0.01, ***p < 0.001.

### Correlation of plasma metabolites with monetary score and preference score

Correlation coefficient of plasma metabolites with monetary score and preference score was shown in Supplementary Tables S6 and S7, respectively. Uric acid showed positive correlation with monetary score in both MDD and HC males, and also showed positive correlation with MDD males’ preference score. These results were consistent with our previous study^36^.

### Correlation of biomarkers with focused preference index

**Figure 2:**
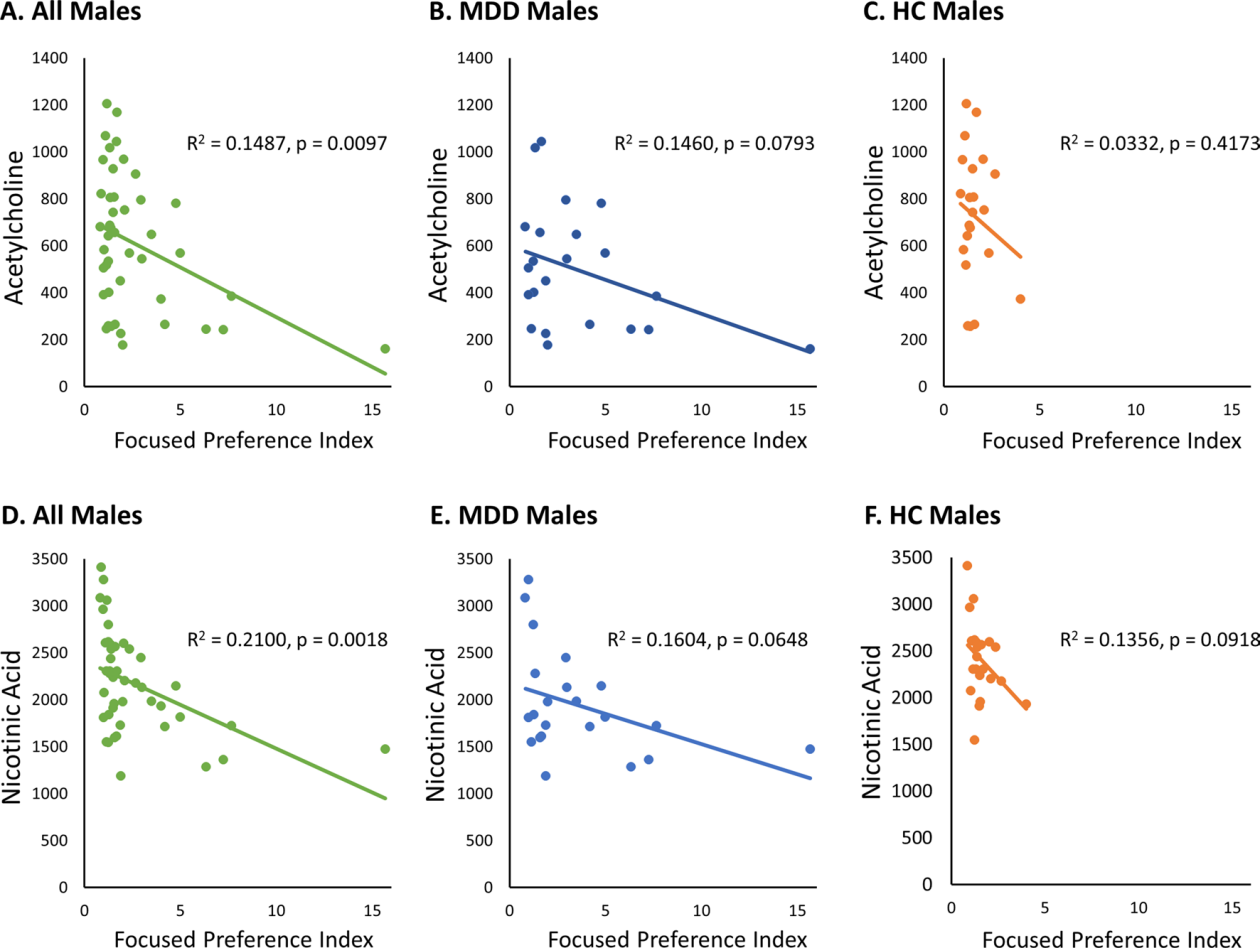
Correlation of biomarker with focused preference index in males. Scatter plot showing negative correlation of focused preference index between plasma acetylcholine (**A**–**C**) and nicotinic acid (**D**–**F**) in MDD and HC males. Linear regression lines with dot plots showing relationship between focused preference index and (**A**) acetylcholine among all males (N = 44), (**B**) acetylcholine among MDD males (N = 22), (**C**) acetylcholine among HC males, (**D**) nicotinic acid among all males (N = 44), (**E**) nicotinic acid among MDD males (N = 22), and (**F**) nicotinic acid among HC males (N = 22). *MDD* major depressive disorder, *HC* healthy controls.

As shown above, we introduced a “focused preference index” to explore deeper traits of preference for others. Thereafter, to investigate whether these blood biomarkers are associated with the focused preference index for the opposite sex, correlation analysis was performed. Interestingly, acetylcholine (Fig. 2A–C) and nicotinic acid (Fig. 2D–F) showed a negative correlation with males’ focused preference index for female photographed partners. On the other hand, these correlations among females for male photographed partners were not observed (data were not shown).

## Discussion

In the present case–control study using PC-based trust game, we have investigated MDD patients’ traits of trusting behaviors and preference for others, and have successfully identified some blood biomarkers relevant to these traits.

### Gender differences in trusting behaviors

In the PC-based trust game, male participants invested more money in partners than female participants. These results are consistent with previous studies^49–51^, suggesting that males tend to show trusting behaviors compared to females. Such male trusting behavior might be partly explained by gender differences and socially accepted norms of behavior which place higher emphasis on male competitiveness and female interpersonal skills and empathy^52,53^. On the other hand, menstrual cycle may have effects in female participants. It is widely known that sex hormones influence emotions and a variety of psychiatric conditions especially depressive symptoms^54^, suggesting that sex hormones also influence social decision-making in females based on each participant’s menstrual cycle. In the present study, we did not ask about the menstrual cycle. Future investigations should be conducted with this point in mind.

### Effects of partners’ appearance and focused preference

Previous studies have investigated the effect of partners’ appearance, which are mainly classified into trustworthy appearance^13,55^ and attractive appearance^56,57^. The latter is thought to be relevant to preference for others, since previous studies targeting autism spectrum disorders and borderline personality disorder reported that attractive appearance has impact on not only trusting behaviors but also preference for others regarding dependability^56,57^. As for the association between depressive symptoms and the attractiveness of others, research using the trust game is limited except for our previous research^19^.

In the present study, we have introduced the focused preference index, which can estimate the “picky” or “choosy” preference tendency. MDD males’ focused preference index for photographed female partners was particularly high (Fig. 1 and Supplementary Table S3). Overall, both MDD patients and HC participants showed more preference for high-attractive partners compared to ordinary-attractive partners. Interestingly, MDD males showed preference for high-attractive female partners about 3.5 times higher to ordinary-attractive females, indicating that depressed males tend to exhibit highly focused (narrowed) preference. This narrowed tendency of depressed males’ preference is likely related to cognitive flexibility. Cognitive flexibility refers to the ability to adapt to unpredictable and complicated surroundings, and plays a crucial role in social cognitive functioning^58,59^. Cognitive flexibility and its related social cognitive functioning such as empathy, theory of mind and facial affect perception are reported to be lowered in depressed patients^60–62^. Lowered cognitive flexibility might impair the ability to approve of others, leading to narrowed (i.e. focused) preference in depressed males. Interpersonal dysfunction including social withdrawal and alienation of others is commonly observed in depressed patients^37,63^, and focused preference possibly promotes such social dysfunction.

On the other hand, MDD males’ high focused preference might facilitate a novel therapeutic approach in MDD. Previous reports have shown that males generally show higher preference and motivation toward attractive opposite-sex faces^64,65^. Attractive faces are easier for visual processing^66^, suggesting that attractive appearance enhances the ability of MDD patients with impaired cognitive functioning or lowered motivation to be attracted. In addition, the ability to experience beauty is hindered by anhedonia (namely, loss of interest/pleasure), but not by depressive feelings per se^67^. Considering such factors, MDD males’ ability to feel opposite-sex attractiveness is likely to be maintained (or not impaired). Furthermore, high focused preference would encourage novel interpersonal therapeutic application for MDD patients. Also, the trust game might be a prospective tool for assessing not only trusting behaviors but also such focused preference. To elucidate this hypothesis, future research is warranted.

### Biomarker analysis

A comparison of plasma metabolites and routine blood biochemical markers results showed significant differences among 33 biomarkers and 4 routine blood biochemical markers in MDD patients. These results partly confirmed our previous studies as to the characterization of depression biomarkers in plasma (i.e. tryptophan and alanine are lower in MDD patients)^26,28^.

Interestingly, nucleic acid-related metabolites such as AMP and GMP were significantly lower and correlated with the preference score in MDD patients. Nucleotides are the building blocks for DNA/RNA synthesis, for which blood cells including lymphocytes are highly required for their proliferation, maturation, and immune function^68^. Nutritionally, it has been established that dietary nucleotides are beneficial to promote the growth of infants and enhance the immune system^69^. Mice fed on a nucleotide-free diet has been shown to be vulnerable to infection, suggesting the reduced levels of nucleotides can affect homeostasis of systemic immunity^68^. The answer of why the nucleotides are lower in plasma with MDD patients remains to be addressed, and we suggest these nucleotides may be the key molecules to unraveling the long-mysterious relationship between depression and immunity.

### Focused preference and blood biomarker

In the present study, we have identified that acetylcholine and nicotinic acid showed a negative correlation with males’ focused preference for photographed females. These results suggest that a decrease (or lack) of acetylcholine and nicotinic acid in the blood may be related to the characteristics of MDD patients who exhibit high focused preference.

A body of research points out the association between acetylcholine and cognitive flexibility. Cognitive flexibility is modulated by the prefrontal cortex and the striatum, and acetylcholine transmission facilitate the cognitive flexibility process in rodents^70^. In addition, deficits in cognitive flexibility in depressed patients are associated with hypoactivity in the medial prefrontal cortex^71–73^. Interestingly, activity in the ventromedial prefrontal cortex is increased when experiencing beauty stimuli including attractive faces^74–77^. These reports suggest that prefrontal cortex is associated not only with cognitive flexibility but also with focused preference in depression. Furthermore, the present result possibly supports the idea that acetylcholine modulates these social aspects. Further research using brain imaging and blood biomarkers is needed to elucidate the role of acetylcholine in focused preference.

Nicotinic acid also showed a negative correlation with males’ focused preference for photographed females. Nicotinic acid is a compound known as niacin (vitamin B_3_)^78^. Niacin deficiency is known to manifest various psychiatric symptoms including psychotic and depressive symptoms^79^. Interestingly, niacin is suggested to be a possible augmentation treatment for schizophrenia^80^. Although the role of nicotinic acid in depression remains unclear, a case report has shown that nicotinic acid treatment was effective in improving depressive symptoms for a patient with bipolar type II disorder^81^. Further research including randomized controlled trials for nicotinic acid nutrient treatment in MDD may help to elucidate the association between nicotinic acid and MDD.

### Focused preference and microglia

By the way, we have reported that oral administration of minocycline, an antibiotic with inhibitory effect of microglial activation, to healthy males alters their trust-related behaviors using a PC-based trust game. We have found that the amount of money administered to photographed females, which increases in the placebo group, does not increase in the minocycline group^34^. Microglia, immune cells in the brain, play crucial roles in inflammation reactions, and microglial activation has been suggested to play important roles in the pathophysiology of a variety of psychiatric conditions including autism, schizophrenia, depression, and suicide^82–86^. We have proposed that suppressing microglial activation is a novel therapeutic target for depression^87–89^. Thus, the present results of focused preference for photographed females may be influenced by microglial activation in depressed males.

Both acetylcholine and nicotinic acid are linked to microglia. Microglia are known to express α7 nicotinic acetylcholine receptor (α7nAchR)^90^, and α7nAchR agonists are known to protect brain inflammatory reactions by acting on microglia^91^. A recent rodent study has shown that lifelong supplementation of choline (precursor for acetylcholine) ameliorates cognitive deficits by attenuating microglia activation via the α7nAchR^92^. Considering the above reports, we interpret the present outcome that lower acetylcholine in depressed males activates microglia, resulting in disturbing cognitive ability and inducing narrowed preference for females. On the other hand, in our previous metabolomic study, we have found that plasma 3-hydroxybutyrate (3HB), also known as β-hydroxybutyrate (BHB), is associated with severity of depression^28^. Recent rodent studies have revealed that 3HB/BHB has antidepressant-like effects^93,94^. Moreover, 3HB/BHB is known to inhibit microglial activation via the hydroxycarboxylic acid receptor 2 (HCAR2, also known as GPR109A)^95^. Interestingly, nicotinic acid (niacin) binds to the HCAR2^96^. Summing up the above references and the present finding, we herein propose that nicotinic acid may regulate human social decision-making (especially preference-related behaviors) by acting on the HCAR2 in microglia. Future translational studies are expected to elucidate these novel hypotheses.

## Limitations

The present case–control study has some limitations. First, our study has recruited relatively small samples, indicating selection bias. In addition, we did not conduct multiple test correction to avoid the risk of false negatives, as the purpose of this study was the global analysis of a number of blood biomarkers as an exploratory study for a future validation study. Despite the relatively small sample size, we have successfully detected several psychological traits and candidate biomarkers. Since candidate biomarkers such as acetylcholine and nicotinic acid have been identified, follow-up studies with greater sample size and multicenter recruitment should be conducted to validate our preliminary findings. Finally, we did not assess body mass index (BMI) and diet conditions in the present study. Levels of some blood biomarkers might be affected by BMI and diet conditions. Future studies should assess BMI and as to whether blood samples are collected under fasting or non-fasting conditions.

## Conclusions

In the present case–control study targeting drug-free MDD patients, we have shown the prospective utility of PC-based trust game for evaluating not only trusting behaviors but also preference for others. We have introduced “focused preference index” reflecting narrowed preference tendency for others, and have revealed that MDD males exhibited high focused (narrowed) preference for females. This MDD males’ focused preference might encourage novel interpersonal therapeutic application. In addition, we have shown that two blood metabolites, acetylcholine and nicotinic acid, were associated with focused preference among MDD males. Focused preference might be observed not only in MDD but also schizophrenia, autism spectrum disorder, and personality disorders, thus our PC-based tool would be useful to understand such aspect in a variety of psychiatric disorders. Larger studies should be conducted to validate our preliminary findings.

## Supplementary information

Supplementary Information.

## Abbreviations

α7nAchR: α7 Nicotinic acetylcholine receptor
AMP: Adenosine monophosphate
BDI-II: Beck Depression Inventory-Second Edition
BHB: β-Hydroxybutyrate
BMI: Body mass index
D-bil: Direct-bilirubin
FDP: Fibrin/fibrinogen degeneration products
Fib: Fibrinogen
GMP: Guanosine monophosphate
HAMD-17: 17-Item Hamilton Rating Scale for Depression
3HB: 3-Hydroxybutyrate
HC: Healthy controls
HCAR2: Hydroxycarboxylic acid receptor 2
HDL-C: High density lipoprotein cholesterol
hsCRP: High sensitivity C-reactive protein
I-bil: Indirect-bilirubin
LC–MS: Liquid chromatography–mass spectrometry
LDL-C: Low density lipoprotein-cholesterol
MDD: Major depressive disorder
SCID: Structured Clinical Interview for DSM-IV-TR
T-bil: Total-bilirubin
TCI-140: 140-Item Temperament and Character Inventory
Total-C: Total-cholesterol
UA: Uric acid
YYS: Yamagishi and Yamagishi’s trust scale
