# Supplementary material for: Plasma acetylcholine and nicotinic acid are correlated with focused preference for photographed females in depressed males: an economic game study
Source: Sci Rep. 2021 Jan 26;11:2199. doi: 10.1038/s41598-020-75115-4 (PMC7838250; doi:10.1038/s41598-020-75115-4)
Supplement: Supplementary file 1 — Supplementary Information. [file 41598_2020_75115_MOESM1_ESM.pdf]

SUPPLEMENTARY INFORMATION

Plasma acetylcholine and nicotinic acid are correlated with focused preference for photographed females in depressed males: an economic game study.

Hiroaki Kubo
Kyushu University

Daiki Setoyama
Kyushu University

Motoki Watabe
Monash University Malaysia

Masahiro Ohgidani
Kyushu University

Kohei Hayakawa
Kyushu University

Nobuki Kuwano
Kyushu University

Mina Sato-Kasai
Kyushu University

Ryoko Katsuki
Kyushu University

Shigenobu Kanba
Kyushu University

Dongchon Kang
Kyushu University

*Takahiro A. Kato
Kyushu University

Table S1. Correlation of monetary score (trusting behaviors) with YYS and TCI-140 sub-scales

	Male		Female	
	MDD	HC	MDD	HC
YYS	0.253[a]	0.603[a]**	0.284[b]	0.465[a]†
TCI-140				
Novelty Seeking	0.022[a]	0.236[b]	-0.156[b]	0.401[a]
Harm Avoidance	-0.138[a]	-0.313[a]	-0.157[b]	-0.421[a]
Reward Dependence	-0.419[a]†	0.485[a]*	-0.267[b]	0.289[a]
Persistence	0.090[a]	0.126[a]	-0.018[b]	-0.044[a]
Self-Directedness	-0.001[a]	0.124[a]	0.243[b]	0.079[a]
Cooperativeness	0.034[a]	0.368[a]†	0.205[b]	0.140[a]
Self-Transcendence	-0.088[b]	0.221[a]	-0.394[b]	0.366[a]

Note: † $p < .10$, * $p < .05$, ** $p < .01$. a: Pearson's product-moment correlation, b: Spearman's rank correlation

Table S2. Correlation of preference score with YYS and TCI-140 sub-scales

	Male		Female	
	MDD	HC	MDD	HC
YYS	0.392[a]†	0.703[a]***	0.677[a]**	0.612[a]*
TCI-140				
Novelty Seeking	-0.035[a]	0.224[b]	-0.047[a]	0.364[a]
Harm Avoidance	-0.016[a]	-0.394[a]†	-0.157[a]	-0.298[a]
Reward Dependence	-0.277[a]	0.577[a]**	0.102[a]	0.186[a]
Persistence	-0.004[a]	0.235[a]	0.195[a]	0.144[a]
Self-Directedness	-0.049[a]	0.399[a]†	0.370[a]	0.034[a]
Cooperativeness	0.122[a]	0.672[a]***	0.509[a]†	0.156[a]
Self-Transcendence	-0.248[b]	0.040[a]	-0.399[a]	0.084[a]

Note: † $p < .10$, * $p < .05$, ** $p < .01$. a: Pearson's product-moment correlation, b: Spearman's rank correlation

Table S3. Scores of focused preference index

	MDD males	HC males	MDD females	HC females
Focused preference for photographed females (*SD*)	3.51 (*3.35*)	1.61 (*0.69*)	1.65 (*0.85*)	1.13 (*0.48*)
Focused preference for photographed males (*SD*)	1.82 (*2.17*)	1.35 (*0.53*)	1.65 (*0.57*)	1.65 (*2.02*)

Note: Focused preference index is defined as mean high attractive/mean ordinary attractive partners' preference score.

Table S4. Group comparison of plasma metabolites in MDD and HC (identified metabolites)

Characteristic[1]	Overall, N = 76	MDD, N = 38	HC, N = 38	p-value
Acetylcarnitine	91894 (45617)	85424 (50939)	98364 (39207)	0.109
**Acetylcholine**	**617 (248)**	**525 (226) ↓**	**709 (236)**	**<0.001*** **
**Aconitic acid**	**4769 (1356)**	**4483 (1308) ↓**	**5056 (1359)**	**0.032***
Adenine	951 (284)	967 (289)	936 (283)	0.319
**Adenosine**	**7526 (2852)**	**6457 (1704) ↓**	**8595 (3352)**	**<0.001*** **
**Adenosine monophosphate**	**17621 (13553)**	**10650 (7802) ↓**	**24593 (14547)**	**<0.001*** **
**Alanine**	**108588 (40941)**	**99181 (38100) ↓**	**117996 (42002)**	**0.022***
2-Aminobutyric acid	17166 (7646)	15922 (7879)	18410 (7299)	0.079†
4-Aminobutyric acid	8015 (2835)	7753 (3090)	8277 (2570)	0.212
5-Amino Levulinic Acid	33510 (14108)	35105 (12114)	31914 (15859)	0.164
**Arginine**	**91841 (71657)**	**54014 (29684) ↓**	**129668 (81167)**	**<0.001*** **
Asparagine	8087 (3024)	7887 (2999)	8287 (3077)	0.284
Aspartic acid	5680 (3671)	5948 (3824)	5413 (3543)	0.265
**Asymmetric dimethylarginine**	**3002 (754)**	**2813 (766) ↓**	**3191 (701)**	**0.014***
**Carnitine**	**66646 (24674)**	**61205 (23678) ↓**	**72086 (24752)**	**0.027***
Choline	13096 (4538)	12732 (5089)	13461 (3946)	0.244
Citric acid	6530 (2863)	6992 (3079)	6068 (2587)	0.080†
Citrulline	36032 (19206)	32955 (13803)	39108 (23190)	0.083†
Creatine	56454 (26410)	55786 (26371)	57122 (26787)	0.414
**Creatinine**	**107225 (39059)**	**97260 (31705) ↓**	**117191 (43397)**	**0.013***
Cysteamine	190 (85)	172 (80)	204 (88)	0.068†
Cysteine	896 (371)	869 (406)	922 (335)	0.267
**Cystine**	**10515 (9817)**	**5208 (3600) ↓**	**15821 (11160)**	**<0.001*** **
**Cytidine**	**541 (196)**	**496 (201) ↓**	**586 (182)**	**0.022***
Cytidine 3',5'-cyclic monophosphate	226 (109)	212 (109)	241 (108)	0.123
Cytosine	500 (208)	499 (183)	502 (233)	0.478
**Dimethylglycine**	**12148 (4892)**	**10777 (3951) ↓**	**13518 (5389)**	**0.007** **
Dopa	310 (154)	319 (156)	301 (154)	0.307
Dopamine	145 (92)	162 (84)	129 (97)	0.061†
Epinephrine	1954 (586)	1969 (538)	1939 (637)	0.415
**Fumaric acid**	**748 (249)**	**813 (251) ↑**	**684 (233)**	**0.011***
Glutamic acid	57601 (26278)	57400 (26551)	57802 (26356)	0.474
**Glutamine**	**558956 (183344)**	**518638 (168131) ↓**	**599275 (191132)**	**0.027***
5-Glutamylcysteine	196 (122)	211 (141)	172 (78)	0.082†
**Glycine**	**7303 (3200)**	**6231 (2915)**	**8376 (3145)**	**0.001** **
**Guanosine**	**3382 (882)**	**3081 (851) ↓**	**3683 (817)**	**0.001** **
**Guanosine monophosphate**	**1712 (1233)**	**1223 (910) ↓**	**2162 (1328)**	**<0.001*** **
Histamine	735 (252)	737 (241)	733 (265)	0.471
**Histidine**	**149209 (55974)**	**130431 (49268) ↓**	**167986 (56561)**	**0.001** **

4-Hydroxyproline	11513 (6257)	10858 (5969)	12168 (6545)	0.182
Hypoxanthine	1399 (938)	1304 (1110)	1494 (729)	0.190
**Inosine**	**860 (1210)**	**628 (386) ↓**	**1091 (1646)**	**0.050***
Isocitric acid	5795 (2455)	6100 (2732)	5490 (2135)	0.141
Isoleucine	171695 (64395)	173452 (60642)	169937 (68716)	0.407
**2-Ketoglutaric acid**	**23543 (3382)**	**21940 (2955) ↓**	**25145 (3029)**	**<0.001*****
Kynurenine	1869 (777)	1781 (801)	1957 (753)	0.163
**Lactic acid**	**97247 (58869)**	**126455 (44543) ↑**	**68039 (57343)**	**<0.001*****
Leucine	193516 (72209)	192084 (66671)	194948 (78230)	0.432
**Lysine**	**600422 (196474)**	**561157 (185821) ↓**	**639687 (201374)**	**0.041***
**Methionine**	**10244 (4710)**	**7639 (3303) ↓**	**12849 (4486)**	**<0.001*****
Methionine sulfoxide	1023 (632)	1125 (726)	921 (511)	0.080†
Niacinamide	2577 (1973)	2506 (2471)	2648 (1332)	0.378
**Nicotinic acid**	**2155 (490)**	**1986 (489) ↓**	**2325 (434)**	**0.001****
**Norepinephrine**	**9866 (3312)**	**8848 (3006) ↓**	**10884 (3327)**	**0.003****
**Ornitine**	**103780 (49164)**	**118005 (40153) ↑**	**89556 (53588)**	**0.005****
**Orotic acid**	**8161 (5675)**	**11336 (6503) ↑**	**4986 (1514)**	**<0.001*****
Phenylalanine	208662 (70134)	197711 (67326)	219612 (72051)	0.088†
Proline	400107 (146069)	395631 (151591)	404582 (142225)	0.396
**Pyruvic acid**	**922 (488)**	**1089 (404) ↑**	**754 (511)**	**0.001****
**Serine**	**48748 (20610)**	**44256 (20273) ↓**	**53241 (20214)**	**0.028***
Serotonin	395 (316)	359 (362)	431 (261)	0.162
**Succinic acid**	**582 (343)**	**710 (389) ↑**	**454 (231)**	**<0.001*****
**Symmetric dimethylarginine**	**3746 (994)**	**3494 (1137) ↓**	**3966 (802)**	**0.025***
Threonine	78889 (29981)	73726 (29222)	84053 (30221)	0.067†
Thymidine	292 (177)	302 (171)	282 (183)	0.309
**Tryptophan**	**72140 (26430)**	**65924 (26140) ↓**	**78356 (25559)**	**0.020***
**Tyrosine**	**30782 (11963)**	**28388 (11153) ↓**	**33175 (12407)**	**0.041***
Uracil	578 (243)	593 (251)	564 (237)	0.302
Uric acid	10204 (4387)	9945 (4059)	10463 (4733)	0.305
Uridine	2815 (1290)	2864 (1342)	2766 (1251)	0.372
Valine	385731 (147036)	368785 (143498)	402677 (150472)	0.159

Note: [1] Statistics presented: mean of signal intensity (SD). Significant p-values are shown in bold type;
† $p < .10$, * $p < .05$, ** $p < .01$, *** $p < .001$.

Table S5. Comparison of routine blood biochemical markers in MDD and HC

Characteristic[1]	Overall, N = 76	HC, N = 38	MDD, N = 38	p-value
Total-C(HDL+LDL) (mg/dL)	181(30)	175(26)	187(34)	0.083†
HDL-C (mg/dL)	64(15)	64(15)	63(15)	0.71
LDL-C (mg/dL)	117(31)	110(24)	124(36)	0.059†
Fib (mg/dL)	227(49)	216(42)	237(53)	0.062†
**FDP (mg/dL)**	**0.17(0.8)**	**0.079(0.49)**	**0.43(1.0)**	**0.031***
**T-Bil (mg/dL)**	**0.69(0.32)**	**0.79(0.33)**	**0.60(0.29)**	**0.008****
**D-Bil (mg/dL)**	**0.23(0.11)**	**0.25(0.11)**	**0.19(0.097)**	**0.005****
**I-Bil (mg/dL)**	**0.47(0.22)**	**0.54(0.23)**	**0.41(0.20)**	**0.011***
UA (mg/dL)	5.22(1.46)	5.31(1.45)	5.14(1.49)	0.61
hsCRP (ng/dL)	426(665)	374(545)	479(774)	0.50

Note: [1]Statistics presented: mean (SD), Significant p-values are shown in bold type; † $p < .10$, * $p < .05$, ** $p < .01$.

Abbreviations: Total-C, total-cholesterol; HDL-C, high density lipoprotein-cholesterol; LDL-C, low density lipoprotein-cholesterol; Fib, fibrinogen; FDP, Fibrin/fibrinogen degradation products; T-Bil, total-bilirubin; D-Bil, direct-bilirubin; I-Bil, indirect-bilirubin; UA, uric acid; hsCRP, high-sensitivity C-reactive protein.

Table S6. Correlation of monetary score with plasma metabolites

	Male		Female	
	MDD	HC	MDD	HC
Acetylcarnitine	0.217	**0.520***	-0.227	-0.065
Acetylcholine	0.366†	0.290	-0.246	-0.082
Aconitic acid	0.101	0.200	-0.170	-0.429†
Adenine	-0.023	-0.294	0.174	0.063
Adenosine	0.012	-0.113	0.023	0.215
Adenosine monophosphate	**0.560****	0.071	-0.197	-0.065
Alanine	**0.495***	0.337	-0.228	-0.012
2-Aminobutyric acid	0.353	0.394†	-0.136	0.156
4-Aminobutyric acid	0.252	0.329	-0.193	-0.105
5-Amino Levulinic Acid	**0.500***	0.218	-0.449†	0.075
Arginine	**0.483***	0.154	-0.453†	-0.163
Asparagine	0.320	**0.491***	-0.255	0.102
Aspartic acid	-0.055	0.173	-0.257	0.152
Asymmetric dimethylarginine	**0.450***	**0.563****	-0.397	-0.065
Carnitine	**0.425***	**0.519***	-0.175	-0.035
Choline	0.232	0.397†	-0.171	0.060
Citric acid	0.310	**0.596****	-0.141	0.173
Citrulline	0.152	0.205	-0.331	0.265
Creatine	0.355	0.288	-0.320	-0.002
Creatinine	0.314	0.383†	-0.319	0.343
Cysteamine	-0.207	0.271	-0.277	-0.238
Cysteine	0.278	0.128	-0.350	-0.158
Cystine	0.394†	0.252	-0.410	-0.065
Cytidine	0.305	0.081	-0.279	0.195
Cytidine 3',5'-cyclic monophosphate	-0.067	**0.428***	0.482†	-0.042
Cytosine	0.060	-0.272	-0.014	0.059
Dimethylglycine	0.333	0.301	-0.117	0.121
Dopa	-0.388†	-0.023	-0.244	-0.185
Dopamine	0.203	0.098	-0.438†	0.094
Epinephrine	0.215	-0.056	0.169	0.013
Fumaric acid	-0.121	0.077	0.184	0.280
Glutamic acid	0.372†	0.074	**-0.500***	0.239
Glutamine	0.348	**0.443***	-0.241	-0.038
5-Glutamylcysteine	-0.094	0.010	0.432†	0.086
Glycine	0.049	0.350	-0.093	0.002
Guanosine	0.311	0.010	-0.409	0.019
Guanosine monophosphate	**0.595****	-0.033	-0.256	-0.094
Histamine	-0.151	-0.258	0.313	**0.540***
Histidine	**0.470***	0.371†	-0.151	0.071

4-Hydroxyproline	0.027	0.117	-0.241	0.012
Hypoxanthine	-0.289	0.136	0.153	0.207
Inosine	0.075	-0.027	-0.256	0.081
Isocitric acid	0.139	**0.485***	-0.205	-0.070
Isoleucine	0.350	0.342	-0.260	-0.178
2-Ketoglutaric acid	0.102	0.208	-0.448†	-0.038
Kynurenine	**0.514***	0.195	-0.091	-0.073
Lactic acid	0.258	0.151	-0.382	0.037
Leucine	0.341	0.350	-0.319	-0.184
Lysine	0.374†	**0.440***	-0.222	-0.035
Methionine	0.414†	**0.489***	0.022	-0.133
Methionine sulfoxide	0.029	0.121	-0.203	0.394
Niacinamide	0.332	0.023	-0.196	-0.190
Nicotinic acid	0.094	0.168	0.315	**-0.532***
Norepinephrine	0.295	0.244	-0.084	-0.096
Ornitine	0.372†	0.304	-0.075	0.082
Orotic acid	-0.296	0.337	0.018	-0.144
Phenylalanine	0.393†	0.390†	-0.269	-0.065
Proline	0.410†	0.101	0.211	0.050
Pyruvic acid	0.325	0.124	-0.409	-0.008
Serine	0.091	0.281	-0.222	-0.014
Serotonin	0.417†	0.037	-0.049	0.060
Succinic acid	-0.236	0.011	-0.140	-0.131
Symmetric dimethylarginine	0.351	0.223	-0.001	0.230
Threonine	0.270	0.223	-0.054	0.023
Thymidine	-0.246	0.264	0.174	-0.040
Tryptophan	0.312	0.328	-0.112	-0.148
Tyrosine	**0.489***	0.340	0.189	0.083
Uracil	-0.035	-0.128	**-0.555***	-0.381
Uric acid	**0.484***	**0.504***	-0.069	0.123
Uridine	**0.445***	**0.461***	-0.190	0.241
Valine	**0.517***	0.336	-0.198	-0.138

Note: Significant p-values are shown in bold type; † *p* < .10, * *p* < .05, ** *p* < .01.

Table S7. Correlation of preference score with plasma metabolites

	Male		Female	
	MDD	HC	MDD	HC
Acetylcarnitine	0.410†	0.220	-0.293	0.046
Acetylcholine	0.314	0.228	-0.297	-0.119
Aconitic acid	0.282	0.016	-0.130	-0.325
Adenine	-0.217	-0.062	0.271	0.433†
Adenosine	-0.125	-0.165	0.043	0.254
Adenosine monophosphate	**0.505***	-0.281	-0.397	-0.057
Alanine	**0.474***	0.336	-0.352	-0.082
2-Aminobutyric acid	0.324	0.157	-0.246	0.146
4-Aminobutyric acid	0.231	0.264	-0.292	-0.084
5-Amino Levulinic Acid	**0.507***	0.392†	-0.461†	0.359
Arginine	0.343	-0.154	-0.196	-0.476†
Asparagine	0.328	0.407†	-0.236	0.050
Aspartic acid	-0.222	0.109	-0.196	0.008
Asymmetric dimethylarginine	0.303	0.389†	**-0.533***	-0.364
Carnitine	0.347	0.320	-0.335	-0.207
Choline	0.259	0.281	-0.343	0.213
Citric acid	0.377†	**0.485***	-0.208	0.255
Citrulline	0.155	0.165	-0.418	0.253
Creatine	0.169	0.200	**-0.604***	-0.260
Creatinine	0.322	0.267	-0.406	**0.520***
Cysteamine	**-0.427***	0.260	-0.268	-0.414
Cysteine	0.238	0.155	-0.153	0.011
Cystine	0.322	-0.060	-0.179	-0.390
Cytidine	**0.494***	-0.091	-0.013	-0.271
Cytidine 3',5'-cyclic monophosphate	-0.012	0.181	0.462†	0.161
Cytosine	0.173	-0.205	0.354	0.098
Dimethylglycine	0.410†	0.114	-0.273	0.081
Dopa	**-0.562****	0.015	-0.010	0.053
Dopamine	0.153	0.306	-0.353	-0.091
Epinephrine	0.021	-0.150	-0.127	0.017
Fumaric acid	-0.108	0.214	0.387	0.310
Glutamic acid	0.391†	-0.035	**-0.506***	0.171
Glutamine	0.402†	0.250	-0.360	-0.220
5-Glutamylcysteine	-0.276	0.004	0.153	-0.029
Glycine	-0.035	0.319	-0.382	0.093
Guanosine	0.330	0.010	-0.253	0.203
Guanosine monophosphate	**0.569****	-0.367†	-0.303	0.048
Histamine	-0.258	-0.317	0.384	0.026
Histidine	0.415†	0.274	-0.252	-0.030

4-Hydroxyproline	-0.145	0.184	-0.327	0.308
Hypoxanthine	-0.262	0.084	0.100	0.323
Inosine	0.045	-0.091	-0.241	0.354
Isocitric acid	0.266	0.361†	-0.328	-0.024
Isoleucine	**0.448***	0.202	-0.405	0.066
2-Ketoglutaric acid	0.044	0.061	-0.218	-0.387
Kynurenine	**0.508***	0.082	-0.194	-0.075
Lactic acid	0.257	0.308	-0.363	0.286
Leucine	**0.432***	0.240	-0.490†	0.019
Lysine	0.397†	0.268	-0.356	-0.177
Methionine	**0.552****	0.390†	-0.166	-0.149
Methionine sulfoxide	0.005	0.205	-0.042	0.216
Niacinamide	0.279	-0.291	-0.006	-0.192
Nicotinic acid	**0.446***	0.267	0.426	-0.285
Norepinephrine	0.313	0.075	-0.285	0.046
Ornitine	0.311	**0.453***	-0.397	0.325
Orotic acid	-0.348	0.246	0.001	0.068
Phenylalanine	**0.433***	0.287	-0.363	-0.030
Proline	0.393†	0.091	-0.014	0.186
Pyruvic acid	0.405†	0.144	**-0.508***	0.368
Serine	0.027	0.251	-0.171	-0.037
Serotonin	0.360	-0.324	-0.231	-0.200
Succinic acid	**-0.465***	0.003	-0.414	0.108
Symmetric dimethylarginine	0.309	0.100	0.142	0.367
Threonine	0.186	0.171	0.043	-0.116
Thymidine	-0.202	0.227	0.031	-0.328
Tryptophan	0.318	0.234	-0.208	-0.176
Tyrosine	**0.525***	0.242	0.047	0.125
Uracil	-0.237	-0.158	-0.474†	-0.052
Uric acid	**0.442***	0.309	-0.089	0.261
Uridine	0.384†	0.405†	-0.388	0.179
Valine	**0.568****	0.209	-0.404	-0.138

Note: Significant p-values are shown in bold type; † p < .10, * p < .05, ** p < .01.
